# Terahertz Detection by Asymmetric Dual Grating Gate Bilayer Graphene FETs with Integrated Bowtie Antenna

**DOI:** 10.3390/nano14040383

**Published:** 2024-02-19

**Authors:** E. Abidi, A. Khan, J. A. Delgado-Notario, V. Clericó, J. Calvo-Gallego, T. Taniguchi, K. Watanabe, T. Otsuji, J. E. Velázquez, Y. M. Meziani

**Affiliations:** 1 Nanotech Group, Facultad de Ciencias, Universidad de Salamanca, 37008 Salamanca, Spain; atif.khan@usal.es (A.K.); juanandn@usal.es (J.A.D.-N.); vito_clerico@usal.es (V.C.); jaime.calvo@usal.es (J.C.-G.); js@usal.es (J.E.V.); 2 National Institute of Material Sciences, 1-1 Namiki, Tsukuba 305-0044, Japan; taniguchi.takashi@nims.go.jp (T.T.); watanabe.kenji.aml@nims.go.jp (K.W.); 3 Research Institute of Electrical Communication, Tohoku University, Sendai 980-8577, Japan; otsuji@riec.tohoku.ac.jp

**Keywords:** 2D materials, graphene, drag effect, dual grating gate, FETs, plasmons, bowtie antenna

## Abstract

An asymmetric dual-grating gate bilayer graphene-based field effect transistor (ADGG-GFET) with an integrated bowtie antenna was fabricated and its response as a Terahertz (THz) detector was experimentally investigated. The device was cooled down to 4.5 K, and excited at different frequencies (0.15, 0.3 and 0.6 THz) using a THz solid-state source. The integration of the bowtie antenna allowed to obtain a substantial increase in the photocurrent response (up to 8 nA) of the device at the three studied frequencies as compared to similar transistors lacking the integrated antenna (1 nA). The photocurrent increase was observed for all the studied values of the bias voltage applied to both the top and back gates. Besides the action of the antenna that helps the coupling of THz radiation to the transistor channel, the observed enhancement by nearly one order of magnitude of the photoresponse is also related to the modulation of the hole and electron concentration profiles inside the transistor channel by the bias voltages imposed to the top and back gates. The creation of local *n* and *p* regions leads to the formation of homojuctions (np, pn or $pp^{+}$) along the channel that strongly affects the overall photoresponse of the detector. Additionally, the bias of both back and top gates could induce an opening of the gap of the bilayer graphene channel that would also contribute to the photocurrent.

## 1. Introduction

The terahertz (THz) region, located between 0.1 and 10 THz, has remained for a long time the only unexplored portion of the electromagnetic (EM) spectrum. The investigation of the THz region has been systematically hindered by the difficulties encountered to generate and detect THz radiation. The lack of practical THz sources able to provide enough power coined the term “terahertz gap”, that can be extended to include the lack of THz detectors. In the present-day the situation of the instrumentation in the THz region is in strong contrast with the more mature available one in the adjacent regions of the EM spectrum, i.e., the infrared (IR) and the microwaves ones.

Over the last 20 years, the THz science has made considerable progress fueled by the extraordinarily wide range of potential applications of the THz radiation [1,2]. Terahertz sensing can be applied in many different fields: astronomy [3], spectroscopy (the maxima of the spectral response of many molecules and solids lies in the THz range; as the THz response of many substances has been found to be stronger and more distinctive than the one found in the microwave, IR and visible spectral ranges, THz spectroscopy can be advantageously used to define fingerprints of these substances) [4], communications (that can benefit of a bandwidth considerably higher than the current communication systems based on microwaves) [5,6,7,8], security (both spectroscopy and imaging to detect concealed objects can be used) [9,10,11], metrology [12], etc. The resolution of THz radiation is limited by light diffraction and its value is similar to the one of the human eye; therefore, THz rays (T-rays) can be used to generate precise images that, additionally, benefit from the unique properties of THz radiation: materials commonly used in packaging for shipping (plastics, cardboard, …) are transparent to T-rays, while opaque in the visible spectrum, allowing the inspection of concealed objects [13]. Additionally, as T-rays can penetrate few millimeters deep the human skin, they can be used to examine subcutaneous tissue for in vivo diagnosis of skin cancer [14].

New emerging 2D materials have shown spectacular properties both for electronic and optoelectronic applications within the terahertz range (detection, emission, modulation, etc.). Graphene was the first 2D material to be fabricated and characterized both optically and electrically. It exhibits unique properties such as exceptionally high values of carrier mobility (in excess of 100,000 cm^2^/V·s) of both electrons and holes, high thermal conductivity, fast relaxation of charge carriers, and high transparency (97.3%) in the visible range of the EM spectrum [15,16,17,18]. Bilayer graphene has attracted big interest due to the extraordinary property of this material that allows the electrical tuning of its energy band-gap from μeV to few hundreds of meV [19]. This property makes bilayer graphene very attractive for the development of terahertz detectors. The methods used for the synthesis of the 2D materials include mainly the mechanical exfoliation, chemical vapor deposition (CVD) and the artificial stacking of new individual layers. Creation of heterostructures that combine graphene with atomically thin crystal layers of other materials has led to considerable improvements of the quality of the so called van der Waals (vdW) heterostructures; eventually, this has allowed the fabrication of devices with excellent performance that can be used in future electrical and optoelectronic applications. Hexagonal boron nitride (h-BN) has proven to be the most suitable material to fabricate high-quality graphene-based heterostructures because of the great similarity between the crystal lattice structures of both materials. Due to its large bandgap (∼7 eV) h-BN can be used as a dielectric to build transistors with insulated gates and, additionally, it can be effectively used to protect graphene layers from degradation induced by environmental contamination.

Encapsulation of graphene by hexagonal boron nitride enables the fabrication of devices with very low disorder allowing the achievement of extremely high values of carrier mobilities in transistor channels [20]. Accordingly, graphene has been used to develop high-quality THz sensors [21,22], emitters [23] and modulators [24,25,26]. The spectral response of the collective oscillations of charge carriers, described as plasmons, in a semiconductor layer or in the channel of a field effect transistor (FET) under EM excitation lies in the THz region. THz response related to the resonant plasmons of bilayer graphene-based field effect transistors (FETs) at low temperature was already reported [27] and explained in terms of the coupling of the incoming THz beam to the FET channel through several methods such as antennas [23,28], non-uniform metallization [29], FETs with an asymmetric design of the source and drain contacts [30], the use of a dc current flowing between the drain and source contacts of a FET to generate asymmetries inside the channel [31], and the use of asymmetric periodic grating gates [32]. THz detectors based on high-electron mobility transistors (HEMTs) with asymmetric dual-grating gates (ADGG) with exceptional high responsivity levels were reported in [33]. The THz response of these transistors is attributed to the spatial modulation of plasmons generated underneath the gates’ fingers. A similar phenomenon may be generated in graphene-based transistors with asymmetric grating gates in which the incident THz beam is converted to propagating plasmons such that the conservation of momentum is ensured [32,34,35].

In this work, an asymmetric dual-grating gate graphene FET (ADGG-GFET) with an integrated bowtie top antenna was succesfully fabricated and used as a terahertz detector. The channel of the fabricated FET is a sheet of bilayer graphene encapsulated between two flakes of h-BN, this double junction heterostructure lies on a SiO_2_/Si wafer in which the doped substrate was used as the back gate (BG) contact of the transistor. The ADGG-GFET was excited at low temperature (4.5K) by three tones terahertz radiation: 0.15 THz, 0.3 THz and 0.6 THz. The mapping of the photocurrent as a function of the bias voltage of the top and back gates showed an enhancement of the photocurrent, up to 8 nA, in comparison with the photocurrent delivered (close to 1 nA) by a similar transistor devoid of antenna under the same measurement conditions. A central result of the present work is that the output current of this new THz detector based on bilayer graphene is modulated by the dc voltages applied to the transistor gates. Accordingly, the response level of the detector can be readily raised through the transistor biasing. This behavior is identified as due to the setting up of two non-uniform electron and hole concentration profiles along the channel. The formation of these non-uniform profiles give rise to the formation of np, pn or $pp^{+}$ junctions in the channel and these regions will eventually determine the THz response of the sensor.

## 2. Materials and Methods

**Figure 1:**
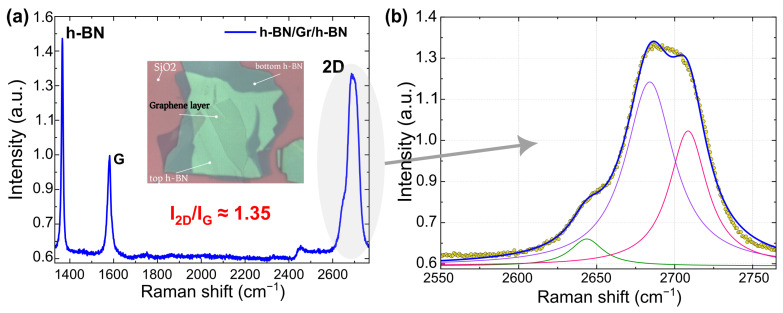
(**a**) Raman spectra of the fabricated vdW heterostructure normalized to the intensity of the G peak. Inset is its optical image. (**b**) The 2D band peak and its Lorentzian fit.

The bilayer graphene flake was obtained by conventional mechanical exfoliation of bulk graphite using Scotch^TM^ tape first and then by folding the tape several times to obtain the graphene flakes. Subsequently, the tape containing the flakes was adhered to a SiO_2_/Si substrate, previously cleaned in an oxygen plasma to remove contaminants, and then annealed for 2 min at 100 °C before slowly removing the tape. This last step allowed the fabrication of flakes with bigger area that are needed for our design. The substrate was a 295 nm thick SiO_2_ layer thermally grown on a 4” highly doped Si wafer. An optical microscope (Leica DM8000) was used to identify the flakes using the high optical transmittance property (∼95% for bilayer). Two relatively thick h-BN layers were obtained from high-quality ultrapure bulk h-BN crystals following the exfoliation process described above without using the oxygen plasma cleaning step as it is not needed. A DektakXT stylus profilometer was used to determine the thickness of both h-BN layers. The h-BN layers were used as the top (∼15 nm thick) and bottom (∼30 nm thick) layers of the h-BN/graphene/h-BN heterostructure. The hot pick-up technique was used for the encapsulation of the graphene layer between the h-BN flakes [36]. First, the top h-BN was picked up by a polymer (Polydimethylsiloxane) on a polycarbonate (PC) film and then transferred on top of the bilayer graphene. The obtained stack PPC/h-BN/graphene was placed in chloroform solution for 24 h to remove and cleanse it of PC. Finally, the same process was repeated to pickup the h-BN/graphene stack and placed on the h-BN bottom layer. The vdW heterostructure (a bilayer graphene sheet sandwiched between two layers of h-BN) has the advantage of protecting graphene from the atmosphere avoiding the degradation of the transistor as stated above [36]. Both the top and bottom h-BN layers were also used to electrically insulate the transistor gates, while the bottom one also serves to reduce the roughness of the back interface of the graphene layer and to reduce the scattering by remote impurities of the charge carriers inside the transistor channel, thus achieving very high values of the carrier mobility in the transistor channel. Raman measurements were realized on the single flakes (graphene and h-BN) as well as on the fabricated vdW double heterostructure using a micro-Raman spectrometer (LabRAM HR Evolution) with a 100× objective using a laser wavelength of 532 nm with a power of 1 mW. Taking the G peak as a reference, the Raman spectrum was normalized and used to evaluate the intensities of the 2D and G peaks ratio $I_{2D}/I_{G}$ which was found to be around 1.35 (Figure 1a). The asymmetrical shape of the 2D peak and the shoulder found around 2650 cm^−1^ indicate that the graphene sheet is a bilayer one [37]. The measured value of the full width at half maximum, FWHM, (Figure 1b) was 56 cm^−1^ which is in good agreement with previously reported results [37].

**Figure 2:**
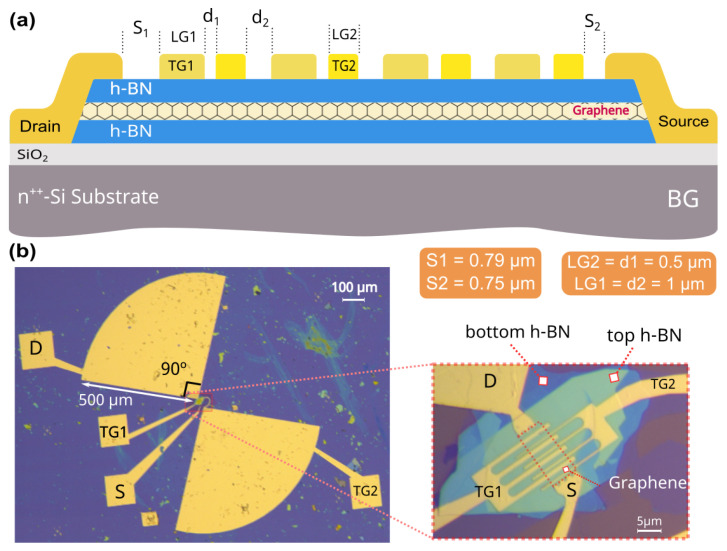
(**a**) Schematic of the cross-section of the fabricated FET showing the h-BN/graphene/h-BN double heterostructure on the SiO_2_/Si substrate, the two top gates, the source and the drain contacts. (**b**) Photograph of the FET with the 90° bowtie antenna, along with a zoomed in photo of the transistor active area showing the asymmetric gratings fingers of both top gates.

Figure 2 shows the design of the ADGG-GFET with the integrated bowtie antenna. The antenna and both the source (S) and the drain (D) metallic contacts were defined by EBL (Electron Beam Lithography) using a polymethyl methacrylate resin, PMMA (6% in chlorobenzene) as the resist, followed by dry etching using an ICP-RIE (Inductively Coupled Plasma Reactive Ion Etching) in SF6 atmosphere (40 SCCM, P = 6 mTorr, P = 75 W at 20 °C) and a subsequent electron beam evaporation of Cr/Au (3.5/60 nm thick). The large distance between the target materials and the sample and the very low pressure of the ICP-RIE chamber (base pressure $10^{-10}$ mbar, process pressure $10^{-8}$ mbar) guarantee a very controllable and homogeneous evaporation process (Chromium evaporation rate: 0.7 A/s, gold evaporation rate: 1.2 A/s). The pyramidal shape of the quasi one dimensional edge contacts (an angle of 40° between the contact and the horizontal plane) ensures that only the S and D metal layers are in contact with the graphene layer which reduces the contact resistance. Later, a second step of EBL along with another evaporation of Cr/Au (5/45 nm thick) was used to fabricate the two independent grating gate structures (TG1 and TG2 in Figure 2a,b) composed of eight fingers in total while a back-gate contact (BG) was defined on the highly doped Si substrate. The geometrical parameters of the asymmetric gratings of the top gates were: $L_{SD}$ = 13 μm, S_1_ = 0.79 μm, S_2_ = 0.75 μm, LG2 = d1 = 0.5 μm and LG1 = d2 = 1 μm (Figure 2a). The top view (Figure 2b) of the device shows the bowtie antenna connected to the drain and to the TG2 of the transistor. The arrangement of the two top gates with the antenna was designed using CST^TM^, a commercial 3D solver of the Maxwell equations, to maximize the photocurrent signal within the sub-THz range. The antenna design was based on the bowtie topology with an angle of 90° and a radius of 500 μm. The antenna geometry was designed to obtain a broadband behavior in the terahertz frequency range under study and to ensure that the spectral response of the antenna will not depend on the frequency if scaled as stated by Rumsey [38]. The maximum operating frequency is limited by the feed terminals, while it’s overall size determines the lowest operation frequency of the antenna. The terminals’ dimensions (approximately equal to the separation between the vertices of the antenna cones) are, in the present case, close to 10 μm and its overall dimension (diameter) is about 1 mm, providing a bandwidth of 1:100, i.e., 2 decades approximately, covering the full range of frequencies under study. The antenna diameter, 1 mm, leads to a resonant coupling of the incident radiation at the lowest operation frequency (0.15 THz) considered in this work. The separation between the antenna feed terminals gives the maximum frequency of operation (around 1 THz). More details on the theoretical basis of the antenna design can be found in [39,40]. The inset of Figure 2b highlights the active part of the transistor showing the asymmetric grating fingers of both top gates of the ADGG-GFET and the vdW heterostructure.

The device was placed inside a variable temperature pulse-tube cryostat with a polyethylene window highly transparent to THz waves. The channel resistance ($R_{\mathrm{SD}}$) of the FET was measured as follows: the internal oscillator of the lock-in amplifier (a Standford Research lock-in model SR860) was used to apply a quasi-dc voltage (1 V, 13 Hz) through a 100 MΩ resistor in series with the channel of the ADGG-GFET ensuring the flow of a quasi-dc current with an intensity of 10 nA. The voltage drop in the channel (between source and drain) was then measured as a function of the gates biases (top and back gates). This electrical characterization of the device versus both gates allowed the extraction of different parameters like the CNP (Charge Neutrality Point), the contact resistances, and the carrier mobility’s.

The ADGG-GFET was then excited, at 4.5 K, by continous-wave terahertz radiation at three tones 0.15, 0.3 and 0.6 THz. We used a TeraSchottky solid-state source with multiplier stages to reach 0.15, 0.3, and 0.6 THz with an emitted power around 6, 3 and 0.8 mW, respectively. This power was measured close to the output of the source by a calibrated pyroelectric detector (THZ 20 from Pyrosensor) with an aperture of 20 mm. The terahertz beam was collimated and focused on the ADGG-GFET using first a 90º off-axis parabolic mirror (with reflected focal distance of 152.4 mm and ∼95% reflectivity) followed by a plano-convex, aspherical TPX (Polymethylpentene) THz lens with a diameter of 50.8 mm, a focal length of 100 mm and transparency around 95% (https://www.tydexoptics.com/pdf/THz_Materials.pdf, accessed on 3 January 2024). The losses of the terahertz power were mainly related to the water vapor absorption in air during the beam propagation. The terahertz beam was electrically modulated at a frequency of 298 Hz that was used as the reference signal for the lock-in amplifier. A low-noise current preamplifier (a Standford Research current preamplifier model SR570) with a sensitivity of 1 μA/V, was placed between the input signal of the lock-in and the drain used to measure the generated photocurrent by the ADGG-GFET. To simplify the analysis of the effect of both top and back gates on the photocurrent, TG1 was kept grounded in all measurements. Accordingly, to simplify the notation, hereafter we will refer to top gate TG2 as TG bearing in mind that TG1 was grounded.

## 3. Results

### 3.1. DC Measurements Results

**Figure 3:**
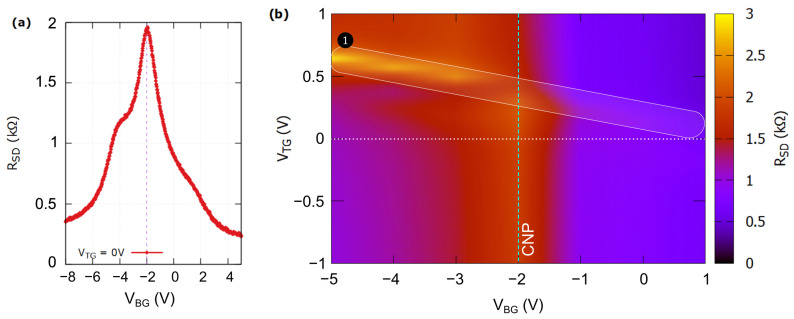
Source-to-drain resistance $R_{SD}$ at 4.5 K (**a**) with respect to $V_{BG}$ where both G1 and G2 were grounded and (**b**), its mapping with respect to $V_{TG}$ and $V_{BG}$.

Figure 3a shows the channel resistance (R_SD_) of the ADGG-GFET versus the back gate voltage (V_BG_) while TG was biased at 0 V. The CNP was observed at $V_{\mathrm{BG}}∼-2$ V, which indicates that the graphene channel is unintentionally n-doped. R_SD_ exhibited two shoulders located approximately at $V_{BG}∼-4$ and +1 V. They are attributed to the influence of the top grating gates that induced asymmetric doping profiles along the channel. A similar behavior has been already reported in [34,41]. The charge carrier mobility was extracted from measurements using the model presented by Gammelgaard et al. in [42]. It was found to be around 23,000 cm^2^/V·s and 17,000 cm^2^/V·s for electrons and holes, respectively.

Figure 3b shows the channel resistance map as a function of both TG and BG bias voltages. While the back gate electrode controls the carrier distribution across the whole channel (including the regions beneath the top gates), the TG competes for control over the electric potential inside the channel and, eventually, modulates the carrier density in the channel’s portions directly below its fingers. For $V_{TG}<$ 0 V, the $R_{SD}$ versus $V_{BG}$ plot exhibits the same behavior observed in Figure 3a with a maximum of R_SD_ at $V_{BG}=-2$ V (CNP indicated with dashed line). When a positive bias was applied to the top gate, a new maximum (with a value of 3 kΩ) is set up for negative voltages of the back gate bias (marked as region 1 in Figure 3b). The shift of the Dirac point entails that, when a negative $V_{BG}$ is applied, specific changes in the concentration levels of electrons or holes in the graphene sheet occur [35,43]. This results in the creation of *n* and *p* type regions along the channel that are induced by the applied bias on both gates. Due to the simultaneous presence of both *n* and *p* regions, interband tunneling may take place between them and, as a consequence, asymmetric bell-shaped $R_{SD}$ plots are observed [44].

### 3.2. THz Measurements Results

**Figure 4:**
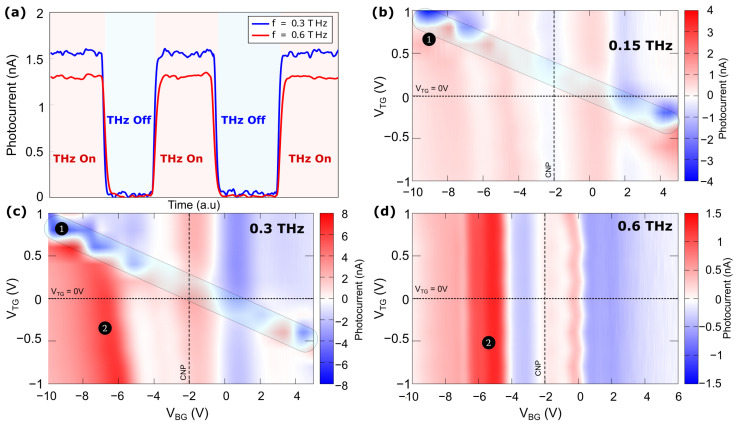
(**a**) Photocurrent vs. time under on/off excitation at 0.3 THz (blue) and 0.6 THz (red) at 4.5 K, while having both top gates grounded. The BG bias was fixed at the maximum intensity of the photocurrent ($V_{BG}=-$6 V for both frequencies). The rectangles were introduced to guide the eye. (**b**–**d**) Photocurrent maps versus top and back gate under excitation at (**b**) 0.15 THz, (**c**) 0.3 THz and (**d**) 0.6 THz.

The ADGG-GFET was cooled down to 4.5 K and excited with three-tones terahertz radiation. The photocurrent induced by the excitation beam was measured at the drain contact using the lock-in technique as mentioned above. Figure 4a shows photocurrent versus time when the THz beam was turned on and off periodically. The signal was obtained under excitation at 0.3 THz (blue) and at 0.6 THz (red) for $V_{BG}=-6$ V and $V_{TG}=0$ V. A photocurrent with a value close to 1.5 nA was measured with a good signal-to-noise ratio (SNR). The SNR is given by: $SNR=10\times \log[P_{Signal}/P_{Noise}$] where $P_{signal}$ and $P_{Noise}$ are the signal and noise power obtained under illumination and dark conditions, respectively. We defined $P_{Signal}=R_{\mathrm{SD}}\times <i_{on}{>}^{2}$ and $P_{Noise}=R_{\mathrm{SD}}\times <i_{off}{>}^{2}$. R_SD_ was assumed to be the same at both conditions (dark and illumination). From the above equation, the new SNR formula is given as:

$$SNR=20\times \log\left[\frac{<i_{on}>}{<i_{off}>}\right]$$

$<i_{on}>$ and $<i_{off}>$ are the root mean square of the measured photocurrent when the beam was switched on and off respectively. They were obtained as $<i>=\frac{1}{N}\sqrt{\sum_{i=0}^{N}i_{i}^{2}}$ where *N* is the number of samples taken from the measurements (Figure 4a) for each condition and frequency. The obtained SNR values were around 36 dB and 37 dB at 0.3 and 0.6 THz, respectively which shows a good level of the photocurrent signal. The generation of the photocurrent is the device response to THz radiation related to the plasmonic nonlinearities in the channel of the FET [32,33,45] and, ultimately, it shows the efficient coupling of the sub-THz wave to the FET channel carried by the ADGG structure and the bowtie antenna [46].

As the multi-gate topology of the transistor consists of three different gates, an analysis of the influence of each independent gate on the THz response of the transistor is necessary. As mentioned before, in this work only the influence of two of the three gates (TG and BG) has been considered. Figure 4b–d shows the photocurrent measured under excitation at three tones (0.15, 0.3 and 0.6 THz) as a function of $V_{TG}$ and $V_{BG}$. Under excitation at 0.15 THz (Figure 4b), the photocurrent shows a maximum of the device response close to ±4 nA along the region 1. The sign changes at the new CNP induced by the TG. Under illumination at 0.3 THz (Figure 4c), a similar behavior was observed with a maximum of the photocurrent of ±8 nA along the region 1. Here, the maximum of the photocurrent remains constant for $V_{BG}$ lower than −6 V (region 2). In both cases (Figure 4b,c), a relative maximum of the photocurrent was also observed around the CNP imposed by the BG voltage ($V_{BG}∼-$2 V), but with an intensity lower than the obtained along the region 1. It can be observed that the magnitude of the photocurrent increased when biases with opposite polarities were applied to TG and BG (along the region 1). The maximum photocurrent levels induced by the 0.15 and 0.3 THz radiation are significantly higher than the ones reported for a similar structure [34] lacking the bowtie antenna. This justifies the use of an antenna to improve the coupling between the THz beam and the channel of the device that gives rise to a strong response of the transistor. In contrast to the behavior observed under excitation at 0.15 and 0.3 THz, the behavior changes radically under excitation at 0.6 THz (Figure 4d) as no significant effect of the TG biasing on the device response was found. The maximum value of the signal was close to ∼1.5 nA at $V_{BG}∼-$6 V.

## 4. Discussion

**Figure 5:**
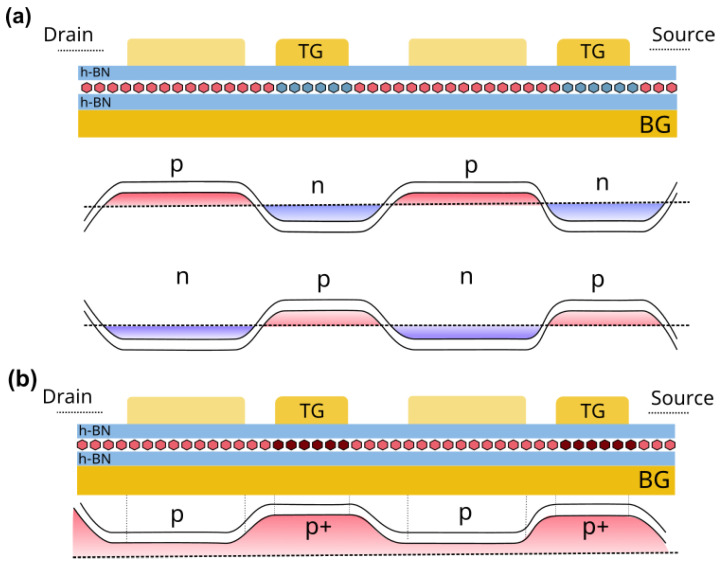
Schematic description of the charge carriers distribution in the channel for (**a**) np regions and (**b**) $pp^{+}$ junctions. Since no bias was applied on TG1, it is assumed that it doesn’t affect the charge carriers.

The maximum photocurrent under excitation at 0.15 & 0.3 THz was obtained around region 1 when the BG voltage was kept below (above) −2 V and a positive (negative) bias voltage was applied to TG (Figure 4b,c). Depending on the biasing conditions of both gates (BG and TG), regions with different carrier’s type (*n* or *p* regions) are created along the channel. The interplay of TG & BG biases determines the electric potential profile in the channel and, consistently, the set-up of regions *n* and *p* with local different carrier concentrations and carrier types (electrons and holes) along the channel. These are: (i) channel regions covered by the TG fingers and whose electric potential is controlled by both TG and BG biases, (ii) the intergate regions (regions between adjacent top gates fingers) in which the electrical potential in the channel portion underneath is essentially controlled by $V_{BG}$. It has been demonstrated [45,47] that the biasing conditions give rise to the so called plasmonic drag effect that shapes the photocurrent response. Figure 5 gives a qualitative description of the internal carrier distributions within the channel that explains the observed improvement of the photocurrent signal. The BG voltage below the CNP (∼−2 V) tends to impose holes (*p*-type region) as majority carriers in the whole channel, while positive voltages on TG induce the formation of *n*-type regions underneath the fingers of the top gate electrode as shown in Figure 5a. As a result, pn regions are created along the channel. If the bias is inverted (above the CNP for the BG and negative values on the TG), np regions will be created. A similar behavior occurs in the case of region 2 (Figure 4) where, in this case, a *p* region is imposed in the channel by the BG and the bias on the TG induced a heavier *p* doping ($p^{+}$) in the regions beneath the TG (Figure 5b). The plasmonic drag effect leads to the generation of the photocurrent as observed in our experiments. In a bilayer graphene channel, an opening of the gap could be induced for the same biasing conditions as previously reported [48,49,50] that would also lead to a behavior very similar to the one observed in the present study.

The photocurrent obtained under excitation at 0.6 THz (Figure 4d) exhibits a behavior distinctively different from the one obtained under illumination at 0.15 & 0.3 THz as no dependence of the photocurrent upon the TG bias was observed. A preliminary interpretation of results points towards a control of the behavior of the device photoresponse by the plasmons generated in the intergate regions of the channel that are essentially controlled by the back gate electrode. This initial interpretation still needs to be confirmed by further measurements and theoretically analyzed to fully understand the origins of the device response.

## 5. Conclusions

A bilayer graphene based ADGG-GFET (asymmetric dual-grating gate Graphene field-effect transistor) with an integrated bowtie antenna was fabricated and characterized as a terahertz detector at three frequencies (0.15, 0.3 and 0.6 THz). Both h-BN and bilayer graphene sheets were obtained by mechanical exfoliation. A van der Waals double junction heterostructure was obtained encapsulating the bilayer graphene sheet between two h-BN layers. The heterostructure was first characterized by Raman spectroscopy (the value of the $I_{2D}/I_{G}$ intensity ratio obtained was close to 1.35) and subsequently used to fabricate the ADGG-GFET. The integration of the bowtie antenna as well as a proper combination of the bias voltages applied to the back and top gates improve the photocurrent response by a factor of 8 in comparison with a similar transistor lacking the bowtie antenna. This enhancement by nearly one order of magnitude of the photoresponse is also related to the modulation of the hole and electron concentration profiles inside the transistor channel by the bias voltages imposed to the top and back gates. The creation of local *n* and *p* regions leads to the formation of pn homojuctions along the channel that strongly affects the overall photoresponse of the detector. Additionally, the bias of both back and top gates could induce an opening of the gap of the bilayer graphene channel that would also contribute to the photocurrent. The present work opens the way for the development of novel THz devices based on the new 2D materials.

## Data Availability

The research data of this works are available upon request to authors.

## Abbreviations

The following abbreviations are used in this manuscript:

ADGG	Asymmetric Dual Grating Gates
BG	Back Gate
CNP	Charge Neutrality Point
D	drain
EBL	Electron Beam Lithography
EM	Electromagnetic
FET	Field Effect Transistor
FWHM	full width at half maximum
S	Source
SNR	Signal to Noise Ratio
TG	Top Gate
THz	Terahertz
vdW	van der Waals
